# Hospital and Institutional News

**Published:** 1916-03-04

**Authors:** 


### HOSPITAL AND INSTITUTIONAL NEWS.

### LORD KNUTSFORD'S ACCIDENT.

Lord Knutsford, whilst crossing Rocks Lane, Barnes, on Tuesday evening, was knocked down by a motor lorry when returning home from a visit to Queen Mary's Convalescent Auxiliary Hospital at Roehampton. Lord Wolverton's car, which was passing, took him to the Putney Hospital, where he was admitted as an in-patient and still lies. The report from the hospital is that Lord Knutsford has been seen by two surgeons, who are of opinion that with care he will make a good recovery. His injuries include lacerated wounds and abrasion of the scalp, bruising, abrasion, and punctured wound of the right leg, a slight abrasion of the left leg, and injury to the knee. No bones were broken. Lord Knutsford is suffering from concussion, but is conscious though drowsy. The latest report is that he has had a fairly good night and is making satisfactory progress. The sympathy of all hospital workers will be extended to Lord and Lady Knutsford, and all will wish that Lord Knutsford's recovery may be rapid and complete.

### THE NEW SWEDISH HOSPITAL.

On Monday last week the Swedish War Hospital for Wounded was opened at 16-18 Paddington Street, W., where accommodation for thirty in-patients has been provided. The hospital has been established by Swedish subscribers and friends of Sweden, and is equipped with a theatre and an *x*-ray installation; the quarters for the staff, though incorporated in the main building, are in an adjoining house. Count Wrangel, who has been Swedish Minister at St. James's for ten years, is hon. chairman, and Dr. K. Westman is resident medical officer. The hospital was accepted by Surgeon-General Russell on behalf of the War Office, and its approval has been received for the reconstruction which has been carried out. Mr. Allan Broman is the honorary superintendent, and the honorary secretaries are Major E. Mossberg and Mr. Louis Zettersten.

### DR. HELEN CAMPBELL'S SALARY WITHHELD.

The action of the staff of the Bradford Infants' Clinic, in consequence of the City Council's refusal to increase, as promised, the salary of the chief medical officer of the department, Dr. Helen Y. Campbell, has ended in sacrifice. Dr. Campbell, in a letter published on Saturday last, stated that she had decided, "in the interests of the cause of infant child-life in Bradford, and whatever the cost to me personally," to withdraw her resignation, "in order that time may prove to both the public and the medical profession the value of the work undertaken." We cannot believe that those members of the Bradford City Council who broke the promise made to Dr. Campbell, and who are responsible for depriving her of a goodly portion of the relatively small salary which is her due, will in the end be supported by the ratepayers, nor proud of their bad faith and wrongdoing.

### THE SUPPLY OF SURGICAL DRESSINGS BY POOR-LAW MEDICAL OFFICERS.

Can a Poor-Law medical officer be required to supply surgical dressings at his own expense to a Poor-Law patient? This question is now under consideration by the Chichester Guardians owing to the refusal of one of their medical officers to do so. The Poor-Law Orders are not clear upon the point. The usual agreement with a medical officer requires him to supply all requisite medicines, except expensive medicines, and the word "medicines" is defined as including all medical and surgical appliances. The Local Government Board has dealt with the matter incidentally in a reply to the Winchcombe Guardians, who asked for a definition of expensive medicines. The Board gave a list of drugs and appliances which might properly be considered expensive, and surgical dressings are included in this list. With the present rise in the price of drugs disputes of this kind will probably arise more frequently than in the past, and will continue until the unsatisfactory system of requiring Poor-Law medical officers to supply medicines is abolished.

### THE SPRINGFIELD MILITARY HOSPITAL.

The Editor of *Truth* recently gave publicity to an accusation of broken pledges directed against the Director-General of the Army Medical Service (Sir A. Keogh) in connection with the arrangements for "nerve-shocked" soldiers. Having availed himself of an invitation to inspect on the spot the Springfield Military Hospital, Wandsworth (around which the contention seems to have arisen), he very emphatically refutes the whole of the criticism which he formerly printed, and incidentally testifies to the efficiency of the Springfield Hospital in a manner which must be highly gratifying to the commandant, Major Worth, who is also superintendent of the neighbouring Wandsworth Asylum, under the Middlesex County Council. An enthusiastic description is given of the hospital buildings themselves, and of the extensive grounds in which they stand; while the system of treatment as Major Worth administers it is also spoken of in the very highest terms of praise. Altogether both the Springfield Hospital and the Army Medical Department get a very excellent testimonial over the incident, which may be regarded as most satisfactorily closed.

### SCOTLAND'S LATEST SANATORIUM.

The new sanatorium, which has been built by the Dundee Town Council at Ashludie, Monifieth, is the latest addition to this class of institution in Scotland. The Convener of the Public Health Committee, Mr. S. G. Fraser, who has taken the principal part in founding and planning the sanatorium, explained that the total cost, £24,000, was made up of £10,000 spent on roads, fencing, and laying out the grounds, £9,000 on new buildings, £3,000 on machinery, and £2,000 on furniture. At the opening ceremony he reminded his hearers that in the long run it was better and cheaper for the community to deal with the housing question than to spend 30s. a week on keeping a patient in a sanatorium. Mr. Thomson, the City Architect, has been engaged on the plans, and Dr. Burgess is the medical officer. Mr. James Wilson, architect to the Local Government Board, congratulated Mr. Thomson on his work, and described the plans as really up to date. The institution includes, of course, a series of revolving chalets in the grounds; and forms part of a general scheme in which the City Dispensary for Home Treatment, and the King's Cross Hospital for Advanced Cases, complete the provision by which incipient cases are to be received at the new Ashludie Sanatorium.

### A TUBERCULOSIS OFFICER FOR THE CITY OF WESTMINSTER.

Unavoidable delays have prevented the maturation of the tuberculosis scheme for the City of Westminster. Negotiations have been proceeding for some time with a view to making use of tuberculosis departments at St. George's Hospital and at Charing Cross Hospital, where patients could be examined and treated or recommended for one of the forms of sanatorium benefit on behalf of the City Council. It has been proposed also to open an office in Pimlico and to arrange for the examination of sputa at Westminster Hospital. It is at the present time no easy matter to find well-qualified tuberculosis officers for new posts, seeing that the majority of such men are liable to be called up for military service. But last Thursday week the Council succeeded in obtaining the services of Dr. A. J. Shinnie, one of the tuberculosis officers under the Surrey County Council. Subject to the approval of the Local Government Board this gentleman will be appointed as temporary tuberculosis officer and assistant medical officer of health for the City at a salary of £500 a year. It may, therefore, be expected that shortly the City of Westminster will fall in line with the majority of the Metropolitan boroughs in retaining the services of a tuberculosis officer.

### THE WELSH MEDICAL SCHOOL.

In the last issue of The Hospital (p. 477), allusion was made, by no means for the first time in these columns, to the action of the Treasury in stifling the birth of the Welsh National Medical School. The circumstances of the dispute between the Welsh University authorities and those at Whitehall have been also put before our readers on previous occasions; and it is gratifying to learn, on the authority of the Parliamentary correspondent of the *Western Daily Mail*, that the urgency of the medical school part of the question is now admitted, and that it is this urgency which has now induced the Government to set up a Royal Commission on University Education in Wales at once, instead of waiting until the end of the war, as hitherto proposed. It is good news to be assured that the importance and urgency of this medical school are at last recognised and admitted. Although a Royal Commission is not exactly a panacea that leads to speed in action or decision, it is permissible to hope that the Commission will take into prior consideration the claims of medical education, and present an *interim* report on them as rapidly as possible before passing on to the longer and more intricate problems of education in general.

### THE CHARTERED ACCOUNTANT AS SECRETARY.

The type of man best suited to the post of hospital secretary admits of endless discussion, and the votes of secretaries in the debate are usually cast in favour of the experience that they themselves brought to the work. Medical, legal, municipal, statistical trainings have all been recommended by the men who have had them, but we note that the Scotch policy of appointing a chartered accountant still holds good, at least in the Glasgow Royal Infirmary. When Mr. Peter Rintoul resigned the secretaryship of that institution last year, after seven years' service, the committee appointed another chartered accountant, Mr. R. Morrison Smith, to succeed him. In doing so their view can be guessed from the fact that they took the opportunity also to merge the previously distinct offices of secretary and cashier. Perhaps the reason why the question who is the best type of man to fill the post of hospital secretary is so popular is that it can never be decided; for the designation "secretary" is not a term of precision. That is why other terms, from "house governor" to the old-fashioned "clerk," persist side by side with it. In Scotland certainly it usually has more specialised meaning than in England; and the proof of this surely is that very few, if any, committees in England would regard a chartered accountant's diploma as an essential qualification.

### PROPOSED REDUCTION OF NOTIFICATION FEES.

Among the suggestions which are stated to be included in the report of the Retrenchments Committee is one for the reduction of the fees at present paid to medical practitioners for the notification of infectious diseases; and it is stated that this particular retrenchment is pressed for with exceptional force. A reduction is contemplated on the present scale of half a crown a case for private practitioners and a shilling a case for institutional officers to a uniform rate of a shilling. We are not aware of what the total annual expenditure under this heading amounts to, but should imagine that £100,000 would cover the bill for the entire country, with something—perhaps a good deal —over; and the reduction will certainly save some thousands a year. For our own part, we cannot see that the present rate of remuneration is more than adequate, considering the service that is rendered and the responsibility attaching to it; and we are at a loss to know why this trivial retrenchment is specially selected. The medical profession might do worse than offer, as an act of charity, to take no fees at all for notifications until the end of the war, reserving the discussion of a just fee for the notification both of infectious diseases

*and of births* until after the war; or, alternatively, might copy the magnanimity of Ministers themselves by graciously condescending to accept War Stock instead of actual cash.

### ARE SANATORIA A WASTE OF MONEY? THE HOUSING PROBLEM AND TUBERCULOSIS.

From such a stronghold of anti-tuberculosis organisation as Edinburgh one would hardly expect to hear serious doubts thrown on the utility of sanatorium treatment. But such is the case, and the report of the City Chamberlain, Mr. Robert Paton, on economy in municipal expenditure, expresses definite views on this matter. Briefly summarised, the conclusion is that a large portion of the money now spent on sanatorium treatment is simply wasted. The opinions of the Medical Officer of Health and the tuberculosis officer are quoted to show that tuberculosis will not be eradicated by spending money in that direction. Both these officers agree that the housing problem is one of the vital points, and it is suggested that if all the money which is at present being poured out on sanatoria had been spent on improving housing conditions, the results would certainly not have been less satisfactory. There can be little difference of opinion as to the effect of dealing boldly with congested and insanitary areas. But the expense would be very great—greater than that of the present anti-tuberculosis measures—and the good results would not be immediately apparent, though it may be confidently assumed that in the next generation the benefit to the community would be sufficiently obvious to satisfy the most exacting ratepayer.

### HOSPITAL OFFICERS BEFORE THE TRIBUNALS.

During the last days of February two at least of the big London hospitals have been represented before the Service Act Tribunals—namely, Guy's and St. George's. The former appealed successfully to have certain students "starred." The latter was less fortunate in its application; the deputy-treasurer asked for the exemption of the resident anæsthetist and of a house surgeon, and these gentlemen received extensions of four months. Considering that until seven or eight years ago the hospital got along very well without a resident anæsthetist, this is generous treatment. Another application on behalf of one of the surgeons, who is unmarried and under forty, was less sympathetically received; one month's extension only was granted. This gentleman would probably have fared much better if he had followed the course of registering his name with the Central Medical War Committee, which is in a much better position to weigh the indispensability to his institution of a hospital medical officer than are the ordinary tribunals set up under the Act. The surgeon in question has, we are told, already served twelve months in the Navy Medical Service, a record which would have obtained for him still further consideration at the hands of the Central Committee. We regard hospital authorities as ill-advised to encourage the appearance of their staffs before the local tribunals in this way, and we think this case will convince waverers of the importance of registering instead with the Central Committee.

### BIRMINGHAM HOSPITALS AND THE WAR OFFICE.

Pressure of news crowded out last week a significant resolution lately passed unanimously at a special meeting of the executive committee of the Birmingham Hospital Saturday Fund. In spite of its length, we give it in full:—

That this meeting of the executive committee of the Birmingham Hospital Saturday Fund, representing nearly 200,000 workers in the city and district, views with alarm the proposals now made further to diminish the medical staffs of the Birmingham hospitals; and, while fully appreciating the need for an adequate Army Medical Service, this committee desires respectfully to point out to the War Office—at a time when the civil hospitals are already working under exceptional pressure and difficulties owing to the depletion of their staffs—the paramount importance of these hospitals at the present time to the inhabitants of Birmingham, which includes so large a proportion of munition workers. The committee is of opinion that any further reduction in the hospital staffs will mean the curtailment of their work, and serious delays and inefficiency in working, at a time when the demands on the hospitals are especially heavy, and when it is of the utmost importance that the hundreds of thousands of workers in Birmingham engaged on munition or Government work should receive, in case of illness or accident, the most skilful medical assistance as promptly as possible. The committee, therefore, respectfully appeals to the War Office to show, if possible, special consideration to the voluntary hospitals of Birmingham and district by not drawing further on their medical staffs unless absolutely necessary.

Sir Hallewell Rogers presided.

### THE STRAIN ON MUNITION WORKERS.

In moving the adoption of this resolution Sir Hallewell Rogers suggested that the War Office might see their way to obtain doctors from country districts rather than reduce the medical staffs of the Birmingham hospitals. He added that the surgical wards were fuller than they had been for thirty years, and that the accidents were more serious than in pre-war days, owing to the pressure of work and the inexperience of many of those engaged on it. Further, the strain on munition workers, he said, no doubt accounted for the fact that even on the medical side the casualty cases were 15 per cent. in excess of the corresponding figures of a year ago. In addition, of course, a large number of beds—one quarter at the General Hospital—were reserved for soldiers; and the already reduced staffs brought them face to face with difficulties. Any intention of further depleting the medical staffs was disconcerting in the extreme. In view of the fact that the health of munition workers is necessary to the output of shells, it is to be hoped that the War Office will rather see what can be done by re-arrangement and better organisation than merely order the depletion of hospital staffs which have already contributed a fair quota.

### THE NEW ARBROATH INFIRMARY.

The new infirmary at Arbroath, which has been built at a cost of £14,700, is practically completed, and the date of its formal opening is under consideration. It is now two years since the erection began, and the completion of the work may be judged from the purchase of furniture and equipment, towards which Mr. Alexander Balfour of Inchrock has given £500, which is to be expended on his behalf by the matron, Miss McElney. The architect is Mr. Hugh Gavin. The temporary infirmary has been situated at Greenbank, with the sale of which the finance committee is now entrusted. An effort is to be made to raise further subscriptions, so that the new infirmary, if possible, may be opened free of debt. To have completed the work under present conditions is satisfactory in itself, and in view of the fact that to do this the relatively small sum of £4,000 only is required, with good management and the taking of proper steps to interest the public, the task should not prove an impossible one.

### WAR WORK AT LIVERPOOL ROYAL INFIRMARY.

It was significant of the intimate connection with war work that has characterised the doings of the Liverpool Royal Infirmary during the past year, that the Treasurer, Mr. Holford Harrison, was absent from the annual meeting last week on military duty. It is interesting to note that, apart from the 525 soldiers who have been admitted, 105 sailors have received treatment in the ward reserved for cases of tropical disease. The number of in-patients was up by 200, and the number of new out-patients, presumably including military cases, has also increased. As the retiring President of the hospital, Lord Salisbury, put it, there was very small loss of income, and a great "increase of business" during the year. The new President, elected at the meeting, was Sir Aubrey Brocklebank, whose namesakes have proved generous benefactors of the hospital for many years. Mr. Ralph Brocklebank, for instance, recently provided the means whereby the committee was able to remodel two of the theatres at the Royal Infirmary. It has been decided to suspend certain bye-laws till six months after the conclusion of peace, so as to empower the committee to suspend the election of an honorary officer, if advisable, and to make any temporary arrangements which may be required. Mr. Wade Deacon, as chairman of the committee, has had a heavy task, but under his direction the hospital has entered on a wider sphere of usefulness.

### ECONOMY IN EXPENSIVE DRUGS.

In two recent communications to medical literature, stress has been laid upon the possibility of economy in two expensive varieties of medical treatment, and the hints thus offered should be useful, especially in institutional practice. Thus it is pointed out in a recent issue of the *Journal of the Royal Army Medical Corps*, as a result of research into the emetine treatment of dysentery, that emetine is a very costly drug; that if it is going to do good its beneficial action is generally quickly apparent; and that it is useless and unwise, as well as wasteful, to subject patients to repeated and long-continued courses of this drug. Many civil hospitals are receiving soldiers, amongst whom dysentery cases are not uncommon; the whole of the article in question is well worth the attention of medical officers who may be in charge of such cases. Then in the *Journal of the American Medical Association* some observations have appeared on the Schick reaction, which is a method used for testing the efficiency of antitoxin immunisation in diphtheria. Amongst other conclusions on this reaction, it is stated that in institutional epidemics it reduces by 50 per cent. the amount of antitoxin used, and so saves a heavy item of expenditure. It does this by showing the attending physician which cases have had enough antitoxin to combat the infection and which have not; thus rendering it unnecessary to err on the safe side by giving very large doses to every patient.

### ANOTHER HEALTH EXPERT.

In a circular which seems to have been fairly liberally distributed through the post, Mr. Eustace Miles has lately informed all whom it may concern that he is "not a qualified medical practitioner," and, in fact, that any who ask his advice about their health do so at their own risk, as it were. If he had been content with that disclaimer there would be little objection in any quarter to his circularising as many people as he likes, but unfortunately there are other statements in his advertisement which are less innocuous. We pass over a series of thinly-veiled sneers at the medical profession; they are unworthy of a man of Mr. Miles' education, but that is all that need be said about them. From following paragraphs it appears that he offers to all and sundry the opportunity of consulting him by correspondence, though a "personal interview" is preferable, and that he has an "expert" to analyse the "blood, urine, etc." Worse still, he copies the methods of the most undesirable advertisers by including a list of dangerous disorders, sufferers from which have regained their health under his treatment. Among these diseases are "kidney troubles" (nature not specified), glycosuria, "liver troubles" (again conveniently vague), neuritis, "lung troubles," and other more or less serious symptoms. All we can say is that in pretending to diagnose and cure from a distance maladies such as these Mr. Miles is undertaking responsibilities which he cannot fulfil.

### A POOR-LAW DEPARTURE AT LEEDS.

The chairman of the Leeds Board of Guardians, Mr. Alfred Hobson, has had the satisfaction of opening the new children's hospital and the operating theatre unit at the Beckett Street institution, Leeds. Standing on pillars to allow a constant passage of fresh air beneath the building, the children's hospital is 160 feet long, comprises three storeys, and accommodates 138 patients. It has cost £9,000, and contains provision for the classification, as well as the isolation, of disease. This important addition forms part of an extension scheme conceived fifteen years ago, the main buildings of which were erected in 1906 at a cost of some £30,000. To complete the original plan a maternity ward and a Lock ward, Mr. Hobson states, are still required. In an interesting speech at the recent opening he pointed out that very few Unions possessed separate children's blocks, but the Leeds Guardians had preferred them. Workhouses had a much bigger future before them. The meaning of "destitution" would be still further qualified; the standard was still being raised, and one day it would be as necessary to have a bacteriologist attached to a Poor-Law infirmary staff as it was to have a dispenser. Mr. H. P. Bagenal, Local Government Board inspector, in congratulating the Guardians on their extensions to the infirmary, attributed the higher standard now prevailing largely to the influence of the Poor-Law Conferences. He pointed out that the extensions had been completed in the nick of time, since after the war many would only be available for absolute necessities.

### THE ECONOMIC ASPECT OF DISPLACING ATTENDANTS BY WOMEN.

The comprehensive paper which we published last week by Dr. George M. Robertson, physician superintendent of Edinburgh Mental Hospital, Morningside, on the advantages of placing female nurses in the male wards of mental hospitals, emphasised conclusions which the war is enforcing upon many institutions hitherto shy of adopting them. Thus the need for making known the Scotch experience of the value of female nurses for male patients in properly administered institutions is still great. For instance, in reply to the Halifax Board of Guardians, the Clerk to the West Riding Asylums Board lately informed them that, in consequence of the shortage of male attendants, the medical superintendent of Wakefield Mental Hospital had appointed female nurses in the male wards. To reassure the Guardians, the clerk then quoted the approval of the Board of Control for this departure. The grounds of this official approval are perhaps worth quoting. The board states that, so far as it can ascertain, "the nurses themselves like it, and the patients benefit from it," that, with proper safeguards, "it generally works well." The one danger is the economic one, which always threatens to curtail salaries and wages whenever women are hurriedly introduced into some new section of the labour market. Institutional staffs are still largely underpaid as a class, and the signs of progress in this direction must not receive a setback through any under-cutting of salaries consequent on the introduction of female "labour" into the male wards of mental hospitals. Luckily mental nursing, especially the female nursing of male mental patients, is a skilled profession, and the adoption of female nursing cannot be recommended as "cheaper," since by it a larger staff is usually required; but the important thing is that the salaries of individual nurse-attendants should not suffer.

### MUNICIPAL MANAGEMENT AT BRADFORD: DOCTOR'S DETAILED CRITICISM.

Taking advantage of the platform provided by the annual meeting of the Bradford Royal Infirmary, Dr. H. G. Campbell, senior hon. physician, discussed the relative economy of voluntary and municipal institutions. He drove his points home from certain figures which he adduced in connection with the infants' clinic that was established by the Corporation in 1912. On inquiry into three years' working, and taking the figures furnished in the official reports of the clinic, the infirmary, and the children's hospital, he found that in 1914 the clinic had 2,488 new out-patients; that the infirmary had 10,139 infant out-patients; while the total out-patient attendances at the clinic numbered 31,193, and at the infirmary 61,306. Then emerged the gravamen of Dr. Campbell's indictment. The cost per case of every infant at the clinic was £4 11s. 9d.; the cost (per out-patient) at the infirmary was 3s. 11d.; the cost per attendance at the clinic was 7s. 3¾d., at the infirmary it was 7¾d. Dr. Campbell further stated that the clinic spent £5,149 on milk, and £2,035 on patent foods, which made the total cost of feeding infant out-patients nearly £7,200. If, said Dr. Campbell, it could be shown that infant mortality had decreased materially the expense would not matter; but, in fact, it had gone up from 116 per 1,000 births in 1909 to 122 in 1914. The deduction he drew was, of course, want of economy in municipal management. His comparison is so detailed, and his figures so definite, that we pause for a reply from the staff of the Bradford Clinic before ourselves passing a judgment upon the apparently invidious comparison which Dr. Campbell has made.

### "HOSPITAL SHIRKERS."

"V. D." writes to the *Times* to inform the public that he has visited many hospitals lately in which he has found a large number of eligible men of military age engaged in doing women's work therein. In one of these hospitals he found seventy young men engaged in washing dishes, polishing floors, carrying patients on stretchers, handing round dressings, giving patients their meals, running messages, and monopolising feminine work. It is evident from "V. D.'s" account that he is alluding to Territorial and military hospitals, and that the young men he refers to are orderlies. We have found in this type of hospital that nearly everywhere there is a difficulty in getting orderlies of any kind, and especially good ones, who are speedily sent by the central authorities from the Territorial hospitals to the Front. It is for the War Office to deal with the point raised. Curiously and helpfully, for so few of the public are technically competent to apprehend the issue, Sir Ernest Hatch, chairman of University College Hospital, in the *Times* points out that every eligible member of the original male staff of his voluntary hospital has enlisted or attested, four have been killed in action, and no new men are engaged without an exemption certificate from the military or naval authorities. The position of University College Hospital in this matter is the position of every well-administered voluntary hospital throughout the country. We welcome the intervention of the chairman of University College Hospital, because it has too long been kept in the background. University College Hospital only needs to have its claims brought prominently before the public to secure for it, as it deserves, all the money and other help it may require from time to time.

### PAYING PATIENTS IN GENERAL WARDS.

At a special meeting of subscribers to the Bridgwater Hospital, the following new rule was adopted, after considerable discussion—namely, that " patients may also be admitted to the public wards by any medical man in the town and district duly qualified in medicine and surgery, on the medical man's recommendation, and subject to the approval of the committee, at a minimum fee of £1 10s. per week, and to the private ward at a minimum fee of £2 2s. per week, and may be attended by the medical man or one of the medical staff on paying his usual or arranged fees for such attendance." While representatives of the friendly societies feared that the new rule would defeat the hospital's object by converting it into a nursing home, Mr. H. J. W. Pollard, chairman of the committee, pointed out that most of the seventeen other hospitals whose experience was consulted had similar rules in force. The aim apparently was rather to regularise an existing practice than to begin a new departure, and one of the medical staff estimated that not more than twelve patients a year would enter the hospital under the new rule. The general principle is that paying patients of this class should be accommodated in pay-wards, but where a hospital is too small to admit of this, the occasional use of a bed in the general wards may be winked at, provided such a bed is not required by the ordinary class of patient. As the new rule expressly inserts the words " subject to the approval of the committee," they, as trustees of the hospital, can easily secure that the new rule is not abused. We appreciate the motive of the proposal to limit the number of beds available for such patients, but it is the committee's duty to do that, and their discretion should be a better check than any fixed number.

### SENSATION-MONGERING IN THE LAY PRESS.

When the leading daily paper of this country descends to printing nonsense such as some which its " medical correspondent " has lately sent in, it is perhaps not surprising that lesser organs of the Press should be easily imposed upon in matters medical. To take a very flagrant case, the Dublin *Evening Mail* published on February 19 a series of reckless mis-statements under five bold headlines, ranging from " Cancer Cure " to " Experts who were Surprised." Stripped of reportorial embellishment and exaggeration, it would appear that an old woman who has had a rodent ulcer for seven years has lately been treated with radium in Dublin, and the ulcer has disappeared. This is doubtless very gratifying to all concerned; but to humbug the public with flaring headlines about " Cancer Cures " is utterly unjustifiable and, in our view, grossly immoral. To begin with, no one can say that this woman is completely cured: some such cases unquestionably are cured permanently, but others presently relapse, and only time can show to which category this patient belongs. The statement that a " board of experts " has examined her and has pronounced her " cured," we decline entirely to credit. It is further stated that before the treatment was begun, specialists had described the case as " incurable ": this again is highly improbable, though it may be that they pronounced it " inoperable "—a very different thing. There are, of course, many ways of treating inoperable cancer, such as *x*-rays, carbonic snow, and actual cautery, besides radium: all of these have been known to effect cure. Then, again, a " cancer expert of international fame " is said to have predicted that the woman would not live five months. Until the *Evening Mail* can produce this " expert," or bring other proof of his alleged statement, we regard this as a falsehood. No man who knew anything about cancer would commit himself to such a statement over a growth on the face of seven years' standing in an old woman. Unfortunately, though the instructed reader can read between the lines of this sort of sensation-mongering nonsense, the general public cannot do so, and takes it for gospel; but newspaper editors have no business to print this sort of thing, and a heavy responsibility rests upon those of them who do so.

### THIS WEEK'S DRUG MARKET.

A further advance in the price of cod-liver oil has taken place since the last report appeared, and, failing the receipt of better news from Norway at an early date, still higher prices may be expected. A cable received from Norway on February 28 quotes a price 15 per cent. in advance of the price quoted at the end of last week. It is, however, even yet too early to form an opinion of the future of this market, although the general opinion prevails that prices will continue high. A good business has been done in menthol at dearer rates, and although there seems little reason to expect any further substantial advance in the near future, no considerable reduction in price is anticipated. The demand for bromides is quieter at the moment, but prices are maintained. Citric acid still has an upward tendency in price, and tartaric acid is distinctly dearer. Considerable business continues to be done in camphor, and higher prices are by no means improbable. The value of cocaine is not quite so firmly maintained. Epsom salts is now freely available. Belladonna root is extremely scarce, and extraordinary prices would have to be paid for any small lots obtainable; belladonna leaves, however, are not so difficult to procure as is the root, but prices are very high. Valerian rhizome is in small supply and fetches a high figure. Liquorice root is very scarce and the value rules accordingly.

